# Optimal achieved blood pressure for patients with stable coronary artery disease

**DOI:** 10.1038/s41598-017-10628-z

**Published:** 2017-08-31

**Authors:** Chin-Chou Huang, Hsin-Bang Leu, Wei-Hsian Yin, Wei-Kung Tseng, Yen-Wen Wu, Tsung-Hsien Lin, Hung-I Yeh, Kuan-Cheng Chang, Ji-Hung Wang, Chau-Chung Wu, Jaw-Wen Chen

**Affiliations:** 1 Department of Medical Education, Taipei Veterans General Hospital, Taipei, Taiwan; 2 Division of Cardiology, Department of Medicine, Taipei Veterans General Hospital, Taipei, Taiwan; 3 Healthcare and Management Center, Taipei Veterans General Hospital, Taipei, Taiwan; 4 Department of Medical Research, Taipei Veterans General Hospital, Taipei, Taiwan; 5 Cardiovascular Research Center, National Yang-MIng University, Taipei, Taiwan; 6 Institute of Pharmacology, National Yang-MIng University, Taipei, Taiwan; 7 Institute of Clinical Medicine, National Yang-Ming University, Taipei, Taiwan; 8 Division of Cardiology, Heart Center, Cheng-Hsin General Hospital, and School of Medicine, National Yang-Ming University, Taipei, Taiwan; 9 Department of Medical Imaging and Radiological Sciences, I-Shou University and Division of Cardiology, Department of Internal Medicine, E-Da Hospital, Kaohsiung, Taiwan; 10 Cardiology Division of Cardiovascular Medical Center and Department of Nuclear Medicine, Far Eastern Memorial Hospital, New Taipei City, Taiwan; 11 School of Medicine, National Yang-Ming University, Taipei, Taiwan; 12 Division of Cardiology, Department of Internal Medicine, Kaohsiung Medical University Hospital and Kaohsiung Medical University, Kaohsiung, Taiwan; 13 Mackay Memorial Hospital, Mackay Medical College, New Taipei City, Taiwan; 14 Division of Cardiology, Department of Internal Medicine, China Medical University Hospital, Taichung, Taiwan; 15 Graduate Institute of Clinical Medical Science, China Medical University, Taichung, Taiwan; 16 Department of Cardiology, Buddhist Tzu-Chi General Hospital, Tzu-Chi University, Hualien, Taiwan; 17 Division of Cardiology, Department of Internal Medicine, National Taiwan University College of Medicine and Hospital, Taipei, Taiwan; 18 Department of Primary Care Medicine, College of Medicine, National Taiwan University, Taipei, Taiwan

## Abstract

We aimed to investigate the ideal achieved blood pressure (BP) in ethnic Chinese patients with stable coronary artery disease (CAD) in Taiwan. A total of 2,045 patients (age 63.5 ± 11.9 years, 1,722 male [84.2%]) with stable CAD who had undergone percutaneous coronary interventions were enrolled. The achieved systolic BP was 130.6 ± 17.7 mmHg and diastolic BP was 74.9 ± 12.0 mmHg. In 12 months, patients with systolic BP < 120 mmHg and systolic BP ≥ 160 mmHg had increased risk of total cardiovascular events when compared to those with systolic BP 120–139 mmHg. In 24 months, patients with systolic BP < 120 mmHg and systolic BP ≥ 160 mmHg had increased risk of total cardiovascular events when compared to those with systolic BP 120–139 mmHg; patients with diastolic BP < 70 mmHg had increased risk of total cardiovascular events when compared to those with diastolic BP 70–79 mmHg. In conclusion, systolic BP < 120 mmHg and ≥160 mmHg or diastolic BP < 70 mmHg is associated with increased cardiovascular events, supporting that the optimal BP control should also be justified for stable CAD in non-western cohorts.

## Introduction

Hypertension plays an important role in cardiovascular morbidities and mortalities. In Prospective Studies Collaboration^1^, blood pressure (BP) is strongly and directly related to vascular and overall mortality starting from at least 115/75 mmHg in one million adults with no previous vascular disease. In a cohort of 1.25 million people initially free from cardiovascular diseases^2^, the lowest risk for cardiovascular diseases was found in individuals with systolic BP 90–114 mmHg and diastolic BP 60–74 mmHg. In the Asia Pacific Cohort Studies Collaboration^3^, BP is an important determinant of the burden of stroke, ischemic heart disease, and total cardiovascular death, with considerable potential benefits when systolic BP lowers to levels of at least 115 mmHg. Recently, the SPRINT trial showed that targeting a systolic BP of less than 120 mmHg in high-risk patients was associated with lower rates of fatal and nonfatal major cardiovascular events and death from any cause^4^. These studies support the importance of aggressive BP control.

However, some studies showed no changes or even increases in cardiovascular events after aggressive BP treatment in specific populations, such as patients with impaired glucose tolerance or diabetes mellitus^5–7^. In the ACCORD trial^7^, intensive treatment (a systolic BP target of less than 120 mmHg) and standard treatment (a target of less than 140 mmHg) in patients with type 2 diabetes produced similar rates of a composite outcome of fatal and nonfatal major cardiovascular events. These findings suggest that BP targets should be different according to underlying comorbidities.

Coronary artery disease (CAD) is a common comorbidity in clinical practice. In the United States, approximately 1 million percutaneous coronary interventions (PCIs) are performed every year, and about 30–45% of them are performed for the management of stable CAD^8, 9^. The recently published CLARIFY registry reported that systolic BP less than 120 mmHg and diastolic BP less than 70 mmHg were each associated with adverse cardiovascular outcomes in patients with stable CAD in western cohorts^10^. This finding raises concerns about possible harmful effects of overly aggressive BP control in CAD patients. Furthermore, the patients in CLARIFY were follow-up for a median of 5 years. However, the optimal achieved BP is still not fully justified for CAD patients worldwide. The aim of this study is to investigate the effects of achieved BP on 12- and 24- month clinical outcomes in a cohort of ethnic Chinese patients with stable CAD in Taiwan.

## Methods

### Study subjects

This is a multicenter study conducted in 9 medical centers in Taiwan^11^. A series of patients were initially evaluated based on history of significant CAD documented on coronary angiogram, history of myocardial infarction as evidenced by 12-lead electrocardiography or hospitalization, or history of angina with ischemic electrocardiography changes or positive response to stress test. Patients were enrolled only if (1) they had received successful PCI with either coronary stenting or balloon angioplasty at least once previously, and (2) they had been stable on medical treatment for at least 1 month before enrollment. Patients were excluded if (1) they had been hospitalized for acute coronary syndrome, acute cerebrovascular events, or other acute cardiovascular events within 3 months before enrollment, (2) they planned to receive further coronary revascularization or interventional procedures for other cardiovascular diseases in the following one year, (3) they had significant malignancy or tumor diseases requiring advanced medical or surgical therapy or both in the following one year, (4) they had other major systemic diseases requiring hospitalization or operation in the following one year, or (5) they were unable or unwilling to be followed up in the following one year. Additionally, patients with life expectancies <6 months (e.g., malignant metastatic neoplasm), or treatment with immunosuppressive agents were also excluded^11^. This study complied with the Declaration of Helsinki. It was approved by the independent ethics committees and independent review boards (IRBs) in each hospital, including Taipei Veterans General Hospital, Cheng-Hsin General Hospital, E-Da Hospital, Far Eastern Memorial Hospital, Kaohsiung Medical University Hospital, Mackay Memorial Hospital, China Medical University Hospital, Buddhist Tzu-Chi General Hospital, and National Taiwan University Hospital, as well as the Joint IRB Ethics Committees Review Boards in Taiwan. All of the patients agreed to participate and signed the study’s informed consent form.

### Baseline data collection

After enrollment, specially trained study nurses and qualified cardiologists collected all data prospectively whenever feasible. Baseline characteristics including risk factors such as history of hypertension, diabetes, smoking and drinking habits as well as medications history were collected by chart review and structured questionnaire.

Body weight and height were recorded in patients without shoes and wearing only light indoor clothes. Body mass index was defined as weight in kilograms divided by the square of height in meters. Waist circumference was measured midway between the iliac crest and the lower-most margin of the ribs. Hip circumference was measured at the maximum circumference of the buttocks as subjects stood with feet placed together. The waist-hip ratio was calculated as 100 × (waist circumference in centimeters/hip circumference in centimeters).

Office BPs were measured at enrollment according to a standardized protocol by a well-trained nurse with an electronic BP monitor in the morning hours after the patients were instructed to sit for 10 minutes in a quiet room. Three consecutive BP measurements were carried out each time. Each measurement was separated by a 30 second pulse measurement. BPs were recorded as the average value of the last two recordings. All analyses were done for systolic BP and diastolic BP separately. Patients were categorized into four groups for each type of BP: systolic BP of <120 mmHg, 120–139 mmHg (reference), 140–159 mmHg, and ≥160 mmHg; and diastolic BP of <70 mmHg, 70–79 mmHg (reference), 80–89 mmHg, and ≥90 mmHg.

### Clinical follow up for adverse cardiovascular events

Each patient was prospectively followed up regularly in individual hospital clinics. After enrollment, follow-up data collection occurred at the time of the outpatient clinic visits, if applicable, and approximately every 3 months for the first year and 6 months after the second year following enrollment. Medication prescriptions were given to each patient at the discretion of the individual treating physician.

During follow-ups, the presence of adverse cardiovascular events were recorded, which included cardiovascular death, nonfatal myocardial infarction, nonfatal stroke, hospitalization for unstable angina, peripheral arterial occlusive disorder, and hospitalization for heart failure. Myocardial infarction was confirmed if ischemic symptoms presented with elevated serum cardiac enzyme levels and/or characteristic electrocardiographic changes. Stroke was confirmed if there was a new neurologic deficit lasting for at least 24 hours with definite imaging evidence of cerebrovascular accident either by magnetic resonance imaging or computed tomography scan. Total cardiovascular events included all the events. Total cardiac events included cardiovascular death, nonfatal myocardial infarction, and hospitalization for unstable angina.

### Statistical Analysis

All baseline characteristics of the patients were expressed as mean ± standard deviation or frequency (percentage). Parametric continuous data between different BP groups were compared by one-way analysis of variance. Categorical variables were analyzed by Chi-Square test or Fisher’s Exact test. Survival analysis was assessed using Kaplan-Meier analysis, with significance based on the log-rank test. A restricted cubic spline smoothing technique was used to interpolate the overall trend of risks through the range of systolic and diastolic BP values, respectively. To assess the association between achieved BP and cardiovascular outcomes, we conducted Cox proportional hazard regression models. In addition to crude hazard ratios (HRs), adjusted HRs were estimated after adjustment for potential confounding factors. HRs of systolic BP for clinical outcomes in 12 months were adjusted for age, gender, body mass index, history of hypertension, history of diabetes, history of ischemic stroke or transient ischemic attack, and concomitant use of B-blockers, calcium channel blockers, and diuretics. HRs of systolic BP for clinical outcomes in 24 months were adjusted for age, gender, body mass index, history of hypertension, history of diabetes, history of ischemic stroke or transient ischemic attack, and concomitant use of anticoagulants, B-blockers, calcium channel blockers, and diuretics. HRs of diastolic BP for clinical outcomes in 12 months and 24 months were adjusted for age, gender, body mass index, history of hypertension, smoking, alcohol drinking, and concomitant use of B-blockers and diuretics. The p-value was two-sided. A p-value < 0.05 was considered statistically significant. All data processing and statistical analyses were performed using Statistical Analysis Software (SAS), version 9.1 (SAS Institute, Cary, North Carolina).

## Results

### Baseline characteristics of the patients

A total of 2,045 patients (age 63.5 ± 11.9 years, 1,722 male [84.2%]) with stable CAD who had undergone percutaneous coronary interventions were enrolled. The achieved systolic BP was 130.6 ± 17.7 mmHg and the achieved diastolic BP was 74.9 ± 12.0 mmHg at enrollment. The past medical histories included hypertension (64.2%), diabetes mellitus (35.8%), ischemic stroke/transient ischemic stroke (2.9%), and heart failure (5.1%). Among these patients, 417 (20.4%) patients had a family history of myocardial infarction. A total of 1,101 patients (53.8%) had a history of smoking, including 656 patients (32.1%) who quit smoking for >1 month, 70 patients (3.4%) who quit smoking for ≤1 month, and 375 patients (18.3%) who were still smoking. A total of 329 patients (16.1%) had a history of alcohol consumption, including 134 patients (6.6%) who drank <1 day/week, 63 patients (3.1%) who drank 1−2 days/week, 55 patients (2.7%) who drank 3–5 days/week, and 77 patients (3.8%) who drank >5 days/week. The drinking amounts were <150 cc/time in 176 patients (8.6%), 150–500 cc/time in 99 patients (4.8%), and >500 cc/time in 54 patients (2.6%). The concomitant medications included anticoagulants (2.7%), antiplatelet (93.0%), angiotensin converting enzyme inhibitors/angiotensin receptor blockers (63.7%), B-blockers (64.7%), calcium channel blockers (39.2%), diuretics (14.0%), nitrate/nicorandil (45.6%), and statins (73.5%). All patients completed the 12-month-follow up, and a total of 1,638 patients completed the 24-month-follow up.

Compared to those with low systolic BP, patients with high systolic BP tended to have histories of hypertension, diabetes mellitus, and ischemic stroke/transient ischemic stroke. They also had higher concomitant use of B-blockers, calcium channel blockers, and diuretics (Tables 1 and 2).

**Table 1:** Baseline characteristics of the patients who completed the 12-month-follow up by achieved systolic blood pressure (n = 2,045).

	SBP < 120 mmHg (n = 530)	SBP = 120~139 mmHg (n = 932)	SBP = 140~159 mmHg (n = 475)	SBP ≥ 160 mmHg (n = 108)	p-value
Age, years	63.1 ± 11.4	63.6 ± 11.8	63.6 ± 12.3	62.7 ± 12.8	0.776
Male, n(%)	458 (86.4%)	781 (83.8%)	395 (83.2%)	88 (81.5%)	0.383
BMI, kg/m^2^	26.0 ± 4.9	26.5 ± 4.2	27.0 ± 3.8	26.6 ± 4.5	0.003
Waist-hip ratio	0.9 ± 0.1	0.9 ± 0.1	0.9 ± 0.1	0.9 ± 0.1	0.189
SBP, mmHg	109.5 ± 7.5	129.3 ± 5.6	147.6 ± 5.5	171.0 ± 12.7	<0.001
DBP, mmHg	65.9 ± 9.3	74.4 ± 9.3	82.3 ± 10.7	90.5 ± 12.8	<0.001
History of hypertension, n(%)	290 (54.7%)	580 (62.2%)	360 (75.8%)	82(75.9%)	<0.001
History of diabetes, n(%)	158 (29.8%)	334 (35.8%)	195 (41.1%)	46 (42.6%)	0.001
History of ischemic stroke/TIA, n(%)	8 (1.5%)	22 (2.4%)	24 (5.1%)	6 (5.6%)	0.002
History of HF, n(%)	31 (5.9%)	40 (4.3%)	29 (6.1%)	5 (4.6%)	0.409
Family history of MI, n(%)	115 (21.7%)	182 (19.5%)	99 (20.8%)	21 (19.4%)	0.776
Smoking, n(%)					0.228
Never	223 (42.1%)	449 (48.2%)	222 (46.7%)	50 (46.3%)	
Quit for >1 month	193 (36.4%)	285 (30.6%)	149 (31.4%)	29 (26.9%)	
Quit for ≤1 month	23 (4.3%)	26 (2.8%)	17 (3.6%)	4 (3.7%)	
Continuous smoking	91 (17.2%)	172 (18.5%)	87 (18.3%)	25 (23.1%)	
Drinking frequency, n(%)					0.333
Never	454 (85.7%)	787 (84.4%)	388 (81.7%)	87 (80.6%)	
<1 day/week	36 (6.8%)	55 (5.9%)	32 (6.7%)	11 (10.2%)	
1–2 days/week	13 (2.5%)	32 (3.4%)	15 (3.2%)	3 (2.8%)	
3–5 days/week	8 (1.5%)	30 (3.2%)	15 (3.2%)	2 (1.9%)	
>5 days/week	19 (3.6%)	28 (3.0%)	25 (5.3%)	5 (4.6%)	
Drinking amounts, n(%)					0.093
No	454 (85.7%)	787 (84.4%)	388 (81.7%)	87 (80.6%)	
<150 cc/time	41 (7.7%)	81 (8.7%)	39 (8.2%)	15 (13.9%)	
150–500 cc/time	21 (4.0%)	47 (5.0%)	27 (5.7%)	4 (3.7%)	
>500 cc/time	14 (2.6%)	17 (1.8%)	21 (4.4%)	2 (1.9%)	
Anticoagulants, n(%)	22 (4.2%)	19 (2.0%)	12 (2.5%)	2 (1.9%)	0.104
Antiplatelet, n(%)	493 (93.0%)	865 (92.8%)	441 (92.8%)	103 (95.4%)	0.801
ACEI/ARB, n(%)	327 (61.7%)	583 (62.6%)	322 (67.8%)	71 (65.4%)	0.166
BB, n(%)	348 (65.7%)	574 (61.6%)	322 (67.8%)	79 (73.2%)	0.023
CCB, n(%)	153 (28.9%)	374 (40.1%)	220 (46.3%)	55 (50.9%)	<0.001
Diuretics, n(%)	74 (14.0%)	110 (11.8%)	83 (17.5%)	19 (17.6%)	0.022
Nitrate/Nicorandil, n(%)	225 (42.5%)	438 (47.0%)	220 (46.3%)	49 (45.4%)	0.400
Statins, n(%)	402 (75.9%)	684 (73.4%)	341 (71.8%)	76 (70.4%)	0.430

ACEI, angiotensin converting enzyme inhibitor; ARB, angiotensin receptor blocker; BB, beta-blocker; BMI, body mass index; CCB, calcium channel blocker; DBP, diastolic blood pressure; HF, heart failure; MI, myocardial infarction; SBP, systolic blood pressure; TIA, transient ischemic attack.

**Table 2:** Baseline characteristics of the patients who completed the 24-month-follow up by achieved systolic blood pressure (n = 1,638).

	SBP < 120 mmHg (n = 410)	SBP = 120~139 mmHg (n = 764)	SBP = 140~159 mmHg (n = 378)	SBP ≥ 160 mmHg (n = 86)	p-value
Age, years	64.1 ± 11.5	63.9 ± 11.8	64.3 ± 12.4	62.5 ± 12.8	0.649
Male, n(%)	348 (84.9%)	645 (84.4%)	317 (83.9%)	70 (81.4%)	0.871
BMI, kg/m^2^	25.9 ± 5.3	26.5 ± 4.3	27.0 ± 3.8	26.6 ± 4.7	0.005
Waist-hip ratio	0.9 ± 0.1	0.9 ± 0.1	1.0 ± 0.1	1.0 ± 0.1	0.087
SBP, mmHg	109.8 ± 7.6	129.2 ± 5.6	147.6 ± 5.5	171.8 ± 13.6	<0.001
DBP, mmHg	65.6 ± 9.7	74.2 ± 9.5	82.3 ± 10.8	90.4 ± 12.9	<0.001
History of hypertension, n(%)	222 (54.2%)	473 (61.9%)	285 (75.4%)	67 (77.9%)	<0.001
History of diabetes, n(%)	124 (30.2%)	283 (37.0%)	157 (41.5%)	40 (46.5%)	0.002
History of ischemic stroke/TIA, n(%)	6 (1.5%)	19 (2.5%)	20 (5.3%)	5 (5.8%)	0.005
History of HF, n(%)	24 (5.9%)	33 (4.3%)	21 (5.6%)	4 (4.7%)	0.650
Family history of MI, n(%)	88 (21.5%)	153 (20.0%)	74 (19.6%)	18 (20.9%)	0.914
Smoking, n(%)					0.358
Never	175 (42.7%)	370 (48.4%)	171 (45.2%)	39 (45.3%)	
Quit for >1 month	148 (36.1%)	228 (29.8%)	123 (32.5%)	22 (25.6%)	
Quit for ≤1 month	16 (3.9%)	22 (2.9%)	11 (2.9%)	4 (4.7%)	
Continuous smoking	71 (17.3%)	144 (18.8%)	73 (19.3%)	21 (24.4%)	
Drinking frequency, n(%)					0.488
Never	353 (86.1%)	646 (84.6%)	313 (82.8%)	71 (82.6%)	
<1 day/week	25 (6.1%)	47 (6.2%)	22 (5.8%)	8 (9.3%)	
1–2 days/week	10 (2.4%)	24 (3.1%)	10 (2.6%)	3 (3.5%)	
3–5 days/week	6 (1.5%)	26 (3.4%)	13 (3.4%)	2 (2.3%)	
>5 days/week	16 (3.9%)	21 (2.7%)	20 (5.3%)	2 (2.3%)	
Drinking amounts, n(%)					0.129
No	352 (85.9%)	646 (84.6%)	311 (82.3%)	71 (82.6%)	
<150 cc/time	34 (8.3%)	63 (8.2%)	27 (7.1%)	10 (11.6%)	
150–500 cc/time	11 (2.7%)	40 (5.2%)	23 (6.1%)	3 (3.5%)	
>500 cc/time	13 (3.2%)	15 (2.0%)	17 (4.5%)	2 (2.3%)	
Anticoagulants, n(%)	20 (4.9%)	16 (2.1%)	8 (2.1%)	2 (2.3%)	0.035
Antiplatelet, n(%)	380 (92.7%)	701 (91.8%)	355 (93.9%)	82 (95.4%)	0.437
ACEI/ARB, n(%)	254 (62.0%)	483 (63.2%)	257 (68.0%)	58 (67.4%)	0.264
BB, n(%)	262 (63.9%)	472 (61.8%)	258 (68.3%)	64 (74.4%)	0.036
CCB, n(%)	124 (30.2%)	310 (40.6%)	180 (47.6%)	45 (52.3%)	< 0.001
Diuretics, n(%)	61 (14.9%)	80 (10.5%)	72 (19.1%)	15 (17.4%)	0.001
Nitrate/Nicorandil, n(%)	185 (45.1%)	364 (47.6%)	172 (45.5%)	41 (47.7%)	0.822
Statins, n(%)	313 (76.3%)	549 (71.9%)	267 (70.6%)	61 (70.9%)	0.267

ACEI, angiotensin converting enzyme inhibitor; ARB, angiotensin receptor blocker; BB, beta-blocker; BMI, body mass index; CCB, calcium channel blocker; DBP, diastolic blood pressure; HF, heart failure; MI, myocardial infarction; SBP, systolic blood pressure; TIA, transient ischemic attack.

Compared to those with low diastolic BP, patients with high diastolic BP tended to be younger, male, hypertensive, and have more smoking and drinking habits. They also had higher concomitant use of B-blockers (Tables 3 and 4).

**Table 3:** Baseline characteristics of the patients who completed the 12-month-follow up by achieved diastolic blood pressure (n = 2,045).

	DBP < 70 mmHg (n = 658)	DBP = 70~79 mmHg (n = 732)	DBP = 80~89 mmHg (n = 419)	DBP ≥ 90 mmHg (n = 236)	p-value
Age, years	67.4 ± 11.9	62.8 ± 11.3	61.5 ± 11.4	58.0 ± 10.9	<0.001
Male, n(%)	516 (78.4%)	633 (86.5%)	370 (88.3%)	203 (86.0%)	<0.001
BMI, kg/m^2^	26.0 ± 5.1	26.3 ± 3.5	27.2 ± 4.3	27.2 ± 4.1	<0.001
Waist-hip ratio	0.9 ± 0.1	0.9 ± 0.1	0.9 ± 0.1	0.9 ± 0.1	0.665
SBP, mmHg	119.2 ± 15.5	129.6 ± 13.0	138.9 ± 13.7	151.1 ± 17.0	<0.001
DBP, mmHg	62.3 ± 6.0	74.1 ± 2.9	83.8 ± 2.9	96.6 ± 6.5	<0.001
History of hypertension, n(%)	395 (60.0%)	469 (64.1%)	280 (66.8%)	168 (71.2%)	0.011
History of diabetes, n(%)	249 (37.8%)	254 (34.7%)	150 (35.8%)	80 (33.9%)	0.583
History of ischemic stroke/TIA, n(%)	12 (1.8%)	24 (3.3%)	16 (3.8%)	8 (3.4%)	0.214
History of HF, n(%)	26 (4.0%)	37 (5.1%)	26 (6.2%)	16 (6.8%)	0.241
Family history of MI, n(%)	125 (19.0%)	143 (19.5%)	87 (20.8%)	62 (26.3%)	0.103
Smoking, n(%)					0.015
Never	322 (48.9%)	349 (47.7%)	181 (43.2%)	92 (39.0%)	
Quit for >1 month	214 (32.5%)	225 (30.7%)	137 (32.7%)	80 (33.9%)	
Quit for ≤1 month	22 (3.3%)	31 (4.2%)	8 (1.9%)	9 (3.8%)	
Continuous smoking	100 (15.2%)	127 (17.3%)	93 (22.2%)	55 (23.3%)	
Drinking frequency, n(%)					0.009
Never	586 (89.1%)	601 (82.1%)	339 (80.9%)	190 (80.5%)	
<1 day/week	26 (4.0%)	59 (8.1%)	29 (6.9%)	20 (8.5%)	
1–2 days/week	14 (2.1%)	22 (3.0%)	15 (3.6%)	12 (5.1%)	
3–5 days/week	15 (2.3%)	22 (3.0%)	14 (3.3%)	4 (1.7%)	
>5 days/week	17 (2.6%)	28 (3.8%)	22 (5.3%)	10 (4.2%)	
Drinking amounts, n(%)					0.006
No	586 (89.1%)	601 (82.1%)	339 (80.9%)	190 (80.5%)	
<150 cc/time	42 (6.4%)	69 (9.4%)	45 (10.7%)	20 (8.5%)	
150–500 cc/time	19 (2.9%)	40 (5.5%)	22 (5.3%)	18 (7.6%)	
>500 cc/time	11 (1.7%)	22 (3.0%)	13 (3.1%)	8 (3.4%)	
Anticoagulants, n(%)	19(2.9%)	18(2.5%)	12(2.9%)	6(2.5%)	0.957
Antiplatelet, n(%)	614(93.3%)	676(92.4%)	390(93.1%)	222(94.1%)	0.803
ACEI/ARB, n(%)	417(63.4%)	469(64.1%)	268(64.0%)	149(63.1%)	0.990
BB, n(%)	402(61.1%)	469(64.1%)	282(67.3%)	170(72.0%)	0.014
CCB, n(%)	250(38.0%)	271(37.0%)	177(42.2%)	104(44.1%)	0.120
Diuretics, n(%)	115(17.5%)	77(10.5%)	58(13.8%)	36(15.3%)	0.003
Nitrate/Nicorandil, n(%)	316(48.0%)	326(44.5%)	190(45.4%)	100(42.4%)	0.408
Statins, n(%)	485(73.7%)	538(73.5%)	310(74.0%)	170(72.0%)	0.955

ACEI, angiotensin converting enzyme inhibitor; ARB, angiotensin receptor blocker; BB, beta-blocker; BMI, body mass index; CCB, calcium channel blocker; DBP, diastolic blood pressure; HF, heart failure; MI, myocardial infarction; SBP, systolic blood pressure; TIA, transient ischemic attack.

**Table 4:** Baseline characteristics of the patients who completed the 24-month-follow up by achieved diastolic blood pressure (n = 1,638).

	DBP < 70 mmHg (n = 539)	DBP = 70~79 mmHg (n = 576)	DBP = 80~89 mmHg (n = 331)	DBP ≥ 90 mmHg (n = 192)	p-value
Age, years	67.8 ± 11.8	63.8 ± 11.3	61.4 ± 11.3	58.1 ± 11.1	<0.001
Male, n(%)	423(78.5%)	496(86.1%)	295(89.1%)	166(86.5%)	<0.001
BMI, kg/m^2^	25.9 ± 5.3	26.3 ± 3.6	27.3 ± 4.6	27.1 ± 3.9	<0.001
Waist-hip ratio	0.9 ± 0.1	0.9 ± 0.1	0.9 ± 0.1	0.9 ± 0.1	0.557
SBP, mmHg	119.5 ± 15.5	130.0 ± 12.6	138.9 ± 13.8	150.8 ± 17.5	<0.001
DBP, mmHg	62.1 ± 6.2	74.1 ± 3.0	83.8 ± 3.0	96.6 ± 6.7	<0.001
History of hypertension, n(%)	320 (59.4%)	377 (65.5%)	211 (63.8%)	139 (72.4%)	0.010
History of diabetes, n(%)	210 (39.0%)	206 (35.8%)	122 (36.9%)	66 (34.4%)	0.609
History of ischemic stroke/TIA, n(%)	11 (2.0%)	16 (2.8%)	15 (4.5%)	8 (4.2%)	0.154
History of HF, n(%)	23 (4.3%)	26 (4.5%)	23 (7.0%)	10 (5.2%)	0.314
Family history of MI, n(%)	99 (18.4%)	112 (19.4%)	72 (21.8%)	50 (26.0%)	0.120
Smoking, n(%)		<0.001			
Never	264 (49.0%)	287 (49.8%)	135 (40.8%)	69 (35.9%)	
Quit for >1 month	175 (32.5%)	169 (29.3%)	111 (33.5%)	66 (34.4%)	
Quit for ≤1 month	18 (3.3%)	25 (4.3%)	4 (1.2%)	6 (3.1%)	
Continuous smoking	82 (15.2%)	95 (16.5%)	81 (24.5%)	51 (26.6%)	
Drinking frequency, n(%)					0.013
Never	483 (89.6%)	474 (82.3%)	268 (81.0%)	158 (82.3%)	
<1 day/week	19 (3.5%)	47 (8.2%)	22 (6.6%)	14 (7.3%)	
1–2 days/week	10 (1.9%)	17 (3.0%)	10 (3.0%)	10 (5.2%)	
3–5 days/week	11 (2.0%)	18 (3.1%)	14 (4.2%)	4 (2.1%)	
>5 days/week	16 (3.0%)	20 (3.5%)	17 (5.1%)	6 (3.1%)	
Drinking amounts, n(%)					0.010
No	483 (89.6%)	473 (82.1%)	268 (81.0%)	156 (81.3%)	
<150 cc/time	32 (5.9%)	53 (9.2%)	35 (10.6%)	14 (7.3%)	
150–500 cc/time	14 (2.6%)	31 (5.4%)	18 (5.4%)	14 (7.3%)	
>500 cc/time	10 (1.9%)	19 (3.3%)	10 (3.0%)	8 (4.2%)	
Anticoagulants, n(%)	17 (3.2%)	14 (2.4%)	12 (3.6%)	3 (1.6%)	0.487
Antiplatelet, n(%)	499 (92.6%)	529 (91.8%)	306 (92.5%)	184 (95.8%)	0.328
ACEI/ARB, n(%)	340 (63.1%)	378 (65.6%)	212 (64.1%)	122 (63.5%)	0.839
BB, n(%)	325 (60.3%)	373 (64.8%)	221 (66.8%)	137 (71.4%)	0.031
CCB, n(%)	211 (39.2%)	221 (38.4%)	140 (42.3%)	87 (45.3%)	0.291
Diuretics, n(%)	93 (17.3%)	59 (10.2%)	46 (13.9%)	30 (15.6%)	0.008
Nitrate/Nicorandil, n(%)	267 (49.5%)	266 (46.2%)	148 (44.7%)	81 (42.2%)	0.275
Statins, n(%)	392 (72.7%)	420 (72.9%)	242 (73.1%)	136 (70.8%)	0.945

ACEI, angiotensin converting enzyme inhibitor; ARB, angiotensin receptor blocker; BB, beta-blocker; BMI, body mass index; CCB, calcium channel blocker; DBP, diastolic blood pressure; HF, heart failure; MI, myocardial infarction; SBP, systolic blood pressure; TIA, transient ischemic attack.

### Clinical outcomes of the patients in 12 months according to systolic blood pressure

In 12 months, both the lowest and highest systolic BP subgroups had higher total cardiovascular events (systolic BP < 120 mmHg vs. 120–139 mmHg vs. 140–159 mmHg vs. ≥160 mmHg = 8.5% vs. 5.5% vs. 7.6% vs.13.0%, p = 0.012), total cardiac events (systolic BP < 120 mmHg vs. 120–139 mmHg vs. 140–159 mmHg vs. ≥160 mmHg = 6.6% vs. 4.4% vs. 6.1% vs. 11.1%, p = 0.021), and hospitalizations for unstable angina (systolic BP < 120 mmHg vs. 120–139 mmHg vs. 140–159 mmHg vs. ≥160 mmHg = 6.0% vs. 3.1% vs. 4.8% vs. 8.3%, p = 0.013) (Table 5).

**Table 5:** Cardiovascular outcomes in systolic and diastolic blood pressure subgroups.

Clinical outcomes in 12 months	SBP < 120 mmHg (n = 530)	SBP = 120~139 mmHg (n = 932)	SBP = 140~159 mmHg (n = 475)	SBP ≥ 160 mmHg (n = 108)	p-value
Total CV events, n(%)	45 (8.5%)	51 (5.5%)	36 (7.6%)	14 (13.0%)	0.012
Total cardiac events, n(%)	35 (6.6%)	41 (4.4%)	29 (6.1%)	12 (11.1%)	0.021
CV death, n(%)	2 (0.4%)	4 (0.4%)	2 (0.4%)	2 (1.9%)	0.224
Nonfatal MI, n(%)	1 (0.2%)	8 (0.9%)	4 (0.8%)	1 (0.9%)	0.458
Unstable angina, n(%)	32 (6.0%)	29 (3.1%)	23 (4.8%)	9 (8.3%)	0.013
Nonfatal stroke, n(%)	1 (0.2%)	2 (0.2%)	1 (0.2%)	0 (0%)	0.972
PAOD, n(%)	4 (0.8%)	4 (0.4%)	3 (0.6%)	1 (0.9%)	0.830
HF hospitalization, n(%)	5 (0.9%)	4 (0.4%)	3 (0.6%)	1 (0.9%)	0.667
**Clinical outcomes in 12 months**	**DBP < 70 mmHg (n = 658)**	**DBP = 70~79 mmHg (n = 732)**	**DBP = 80~89 mmHg (n = 419)**	**DBP ≥ 90 mmHg (n = 236)**	**p-value**
Total CV events, n(%)	56 (8.5%)	44 (6.0%)	24 (5.7%)	22 (9.3%)	0.101
Total cardiac events, n(%)	44 (6.7%)	35 (4.8%)	21 (5.0%)	17 (7.2%)	0.297
CV death, n(%)	3 (0.5%)	3 (0.4%)	2 (0.5%)	2 (0.9%)	0.865
Nonfatal MI, n(%)	1 (0.2%)	6 (0.8%)	4 (1.0%)	3 (1.3%)	0.205
Unstable angina, n(%)	40 (6.1%)	26 (3.6%)	15 (3.6%)	12 (5.1%)	0.098
Nonfatal stroke, n(%)	1 (0.2%)	1 (0.1%)	1 (0.2%)	1 (0.4%)	0.834
PAOD, n(%)	7 (1.1%)	3 (0.4%)	1 (0.2%)	1 (0.4%)	0.268
HF hospitalization, n(%)	4 (0.6%)	5 (0.7%)	1 (0.2%)	3 (1.3%)	0.460
**Clinical outcomes in 24 months**	**SBP < 120 mmHg (n = 410)**	**SBP = 120~139 mmHg (n = 764)**	**SBP = 140~159 mmHg (n = 378)**	**SBP ≥ 160 mmHg (n = 86)**	**p-value**
Total CV events, n(%)	64 (15.6%)	76 (10.0%)	47 (12.4%)	20 (23.3%)	0.001
Total cardiac events, n(%)	48 (11.7%)	65 (8.5%)	38 (10.1%)	17 (19.8%)	0.008
CV death, n(%)	3 (0.7%)	5 (0.7%)	2 (0.5%)	2 (2.3%)	0.350
Nonfatal MI, n(%)	2 (0.5%)	11 (1.4%)	8 (2.1%)	3 (3.5%)	0.099
Unstable angina, n(%)	43 (10.5%)	49 (6.4%)	28 (7.4%)	12 (14.0%)	0.016
Nonfatal stroke, n(%)	2 (0.5%)	2 (0.3%)	1 (0.3%)	1 (1.2%)	0.573
PAOD, n(%)	5 (1.2%)	4 (0.5%)	4 (1.1%)	1 (1.2%)	0.591
HF hospitalization, n(%)	9 (2.2%)	5 (0.7%)	4 (1.1%)	1 (1.2%)	0.135
**Clinical outcomes in 24 months**	**DBP < 70 mmHg (n = 539)**	**DBP = 70~79 mmHg (n = 576)**	**DBP = 80~89 mmHg (n = 331)**	**DBP ≥ 90 mmHg (n = 192)**	**p-value**
Total CV events, n(%)	85(15.8%)	56(9.7%)	38(11.5%)	28(14.6%)	0.016
Total cardiac events, n(%)	64(11.9%)	46(8.0%)	35(10.6%)	23(12.0%)	0.144
CV death, n(%)	5(0.9%)	3(0.5%)	2(0.6%)	2(1.0%)	0.810
Nonfatal MI, n(%)	3(0.6%)	8(1.4%)	8(2.4%)	5(2.6%)	0.075
Unstable angina, n(%)	56(10.4%)	35(6.1%)	25(7.6%)	16(8.3%)	0.068
Nonfatal stroke, n(%)	3(0.6%)	1(0.2%)	1(0.3%)	1(0.5%)	0.733
PAOD, n(%)	9(1.7%)	3(0.5%)	1(0.3%)	1(0.5%)	0.093
HF hospitalization, n(%)	9(1.7%)	6(1.0%)	1(0.3%)	3(1.6%)	0.297

CV, cardiovascular; DBP, diastolic blood pressure; HF, heart failure; MI, myocardial infarction; PAOD, peripheral artery occlusive disease; SBP, systolic blood pressure.

**Figure 1:**
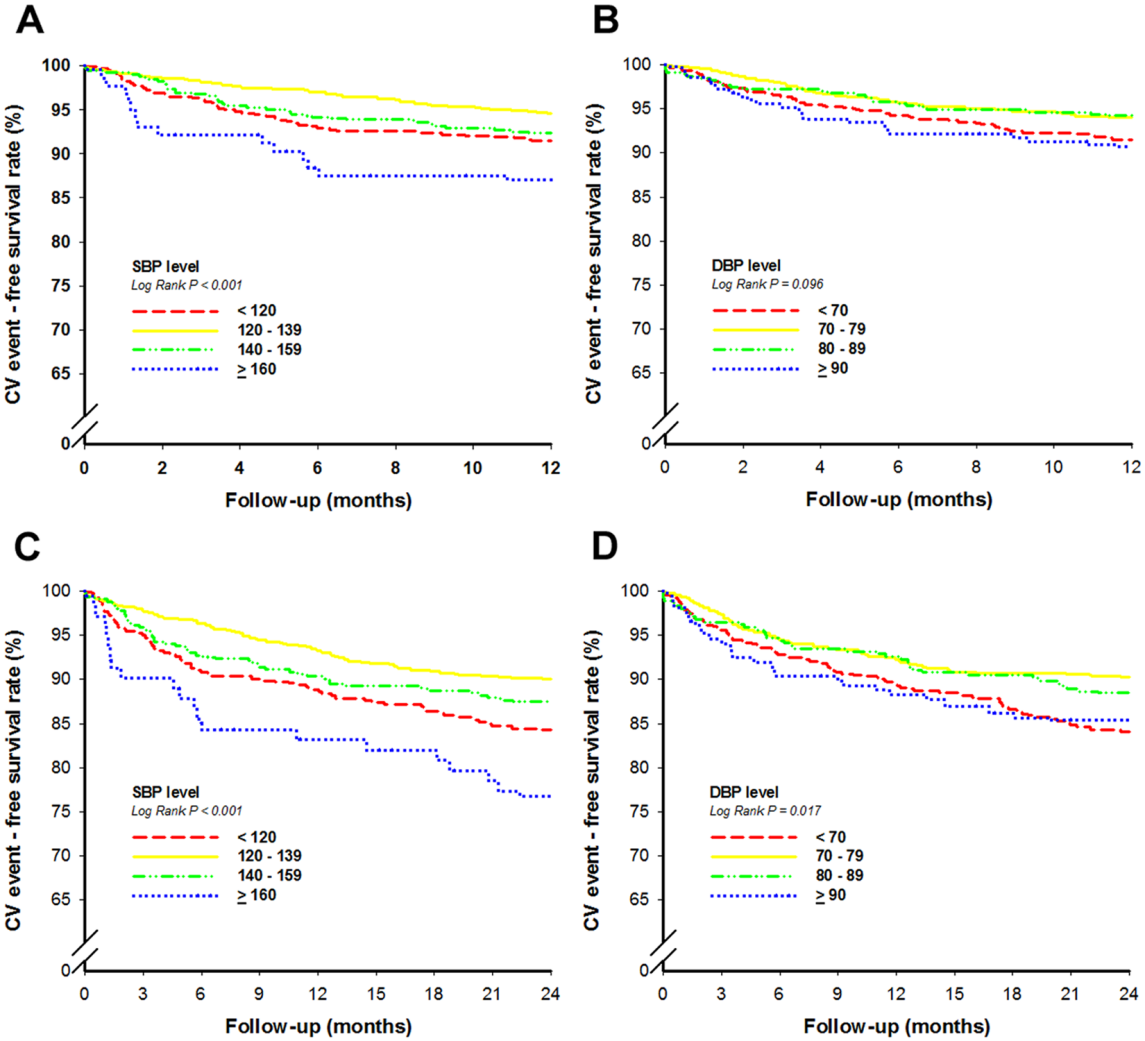
Kaplan-Meier curves of outcomes associated with blood pressure (BP) in patients with coronary artery disease. Shown were rates of cardiovascular events in (**A**) systolic BP subgroups in 12 months, (**B**) diastolic BP subgroups in 12 months, (**C**) systolic BP subgroups in 24 months, and (**D**) diastolic BP subgroups in 24 months. The p-values were calculated with the log-rank test. CV = cardiovascular, DBP = diastolic blood pressure, SBP = systolic blood pressure.

Kaplan-Meier survival plot showed that patients with systolic BP ≥ 160 mmHg had the highest total cardiovascular events, followed by patients with systolic BP < 120 mmHg and then patients with systolic BP 140–159 mmHg; patients with systolic BP 120–139 mmHg had the lowest total cardiovascular events (p < 0.001) **(**Fig. 1A **)**.

**Figure 2:**
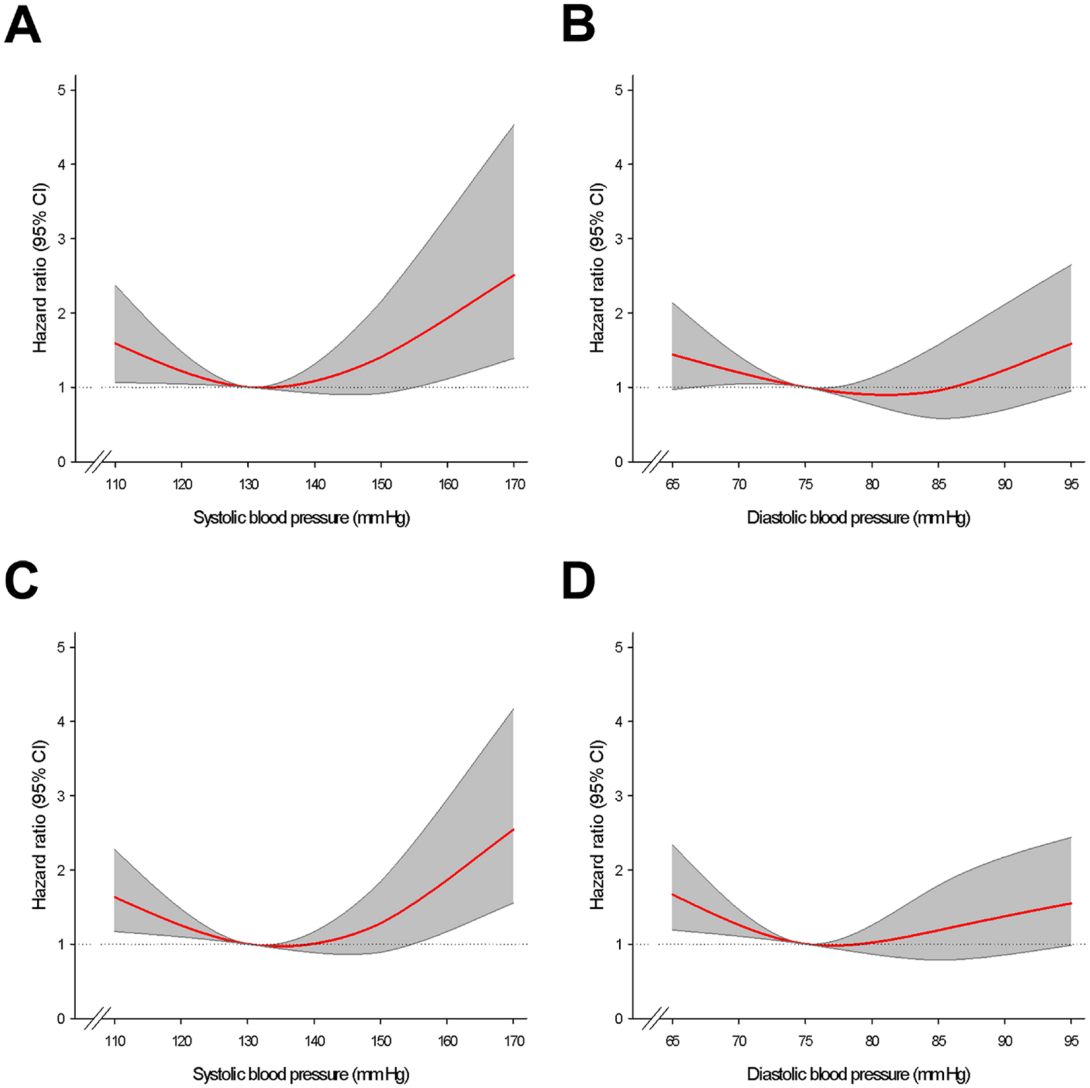
Restricted cubic splines of (**A**) cardiovascular events in 12 months versus average systolic BP, (**B**) cardiovascular events in 12 months versus average diastolic BP, (**C**) cardiovascular events in 24 months versus average systolic BP, and (**D**) cardiovascular events in 24 months versus average systolic BP. CI = confidence interval.

A J-shaped curve was shown for the occurrence of the total cardiovascular events, with increased risk at low and high systolic BP values **(**Fig. 2A **)**.

Cox regression showed that patients with systolic BP < 120 mmHg (hazard ratio [HR], 1.591; 95% confidence interval [CI], 1.065–2.375, p = 0.023) and systolic BP ≥ 160 mmHg (HR, 2.511; 95% CI, 1.390–4.535, p = 0.002) had increased risk of total cardiovascular events compared to those with systolic BP 120–139 mmHg. Multivariate analysis showed that patients with systolic BP < 120 mmHg (HR, 1.640; 95% CI, 1.094–2.457, p = 0.017) and systolic BP ≥ 160 mmHg (HR, 2.377; 95% CI, 1.307–4.322, p = 0.005) had increased risk of total cardiovascular events compared to those with systolic BP 120–139 mmHg (Table 6).

**Table 6:** Crude and adjusted hazard ratios for systolic and diastolic blood pressure subgroups.

	Crude HR (95% CI)	p-value	Adjusted HR (95% CI)	p-value
**Total cardiovascular events in 12 months**				
Systolic blood pressure				
<120 mmHg	1.591 (1.065 − 2.375)	0.023	1.640 (1.094–2.457)	0.017
120~139 mmHg	1		1	
140~159 mmHg	1.407 (0.919–2.156)	0.117	1.353 (0.879–2.083)	0.170
≥160 mmHg	2.511 (1.390–4.535)	0.002	2.377 (1.307–4.322)	0.005
Diastolic blood pressure				
<70 mmHg	1.441 (0.971–2.139)	0.070	1.346 (0.898–2.016)	0.150
70~79 mmHg	1		1	
80~89 mmHg	0.956 (0.581–1.572)	0.860	0.922 (0.560–1.521)	0.752
≥90 mmHg	1.588 (0.952–2.649)	0.077	1.476 (0.879–2.477)	0.141
**Total cardiovascular events in 24 months**				
Systolic blood pressure				
<120 mmHg	1.634 (1.172–2.278)	0.004	1.648 (1.177–2.308)	0.004
120~139 mmHg	1		1	
140~159 mmHg	1.280 (0.890–1.841)	0.184	1.265 (0.875–1.830)	0.212
≥160 mmHg	2.546 (1.556–4.167)	<0.001	.518 (1.528–4.149)	<0.001
Diastolic blood pressure				
<70 mmHg	1.671 (1.192–2.341)	0.003	1.590 (1.125–2.247)	0.009
70~79 mmHg	1		1	
80~89 mmHg	1.187 (0.786–1.792)	0.415	1.135 (0.749–1.718)	0.551
≥90 mmHg	1.550 (0.985–2.440)	0.058	1.418 (0.894–2.251)	0.138

CI = confidence interval, HR = hazard ratio. HRs of systolic blood pressure for clinical outcomes in 12 months were adjusted for age, male, body mass index, history of hypertension, history of diabetes, history of ischemic stroke or transient ischemic attack, and concomitant use of B-blockers, calcium channel blockers, and diuretics. HRs of systolic blood pressure for clinical outcomes in 24 months were adjusted for age, male, body mass index, history of hypertension, history of diabetes, history of ischemic stroke or transient ischemic attack, and concomitant use of anticoagulants, B-blockers, calcium channel blockers, and diuretics.HRs of diastolic blood pressure for clinical outcomes in 12 months and 24 months were adjusted for age, male, body mass index, history of hypertension, smoking, alcohol drinking, and concomitant use of B-blockers and diuretics.

### Clinical outcomes of the patients in 12 months according to diastolic blood pressure

In 12 months, the clinical outcomes were similar in the 4 diastolic BP subgroups (Table 5). Kaplan-Meier survival plot showed no significant differences between the 4 diastolic BP subgroups **(**Fig. 1B **)**. Although a J-shaped curve was shown for the occurrence of the total cardiovascular events **(**Fig. 2B **)**, Cox regression showed that patients in the 4 diastolic BP subgroups had similar risks of total cardiovascular events (Table 6).

### Clinical outcomes of the patients in 24 months according to systolic blood pressure

In 24 months, both the lowest and highest systolic BP subgroups had higher total cardiovascular events (systolic BP < 120 mmHg vs. 120–139 mmHg vs. 140–159 mmHg vs. ≥160 mmHg = 15.6% vs. 10.0% vs. 12.4% vs. 23.3%, p = 0.001), total cardiac events (systolic BP < 120 mmHg vs. 120–139 mmHg vs. 140–159 mmHg vs. ≥160 mmHg = 11.7% vs. 8.5% vs. 10.1% vs. 19.8%, p = 0.008), and hospitalizations for unstable angina (systolic BP < 120 mmHg vs. 120–139 mmHg vs. 140–159 mmHg vs. ≥160 mmHg = 10.5% vs. 6.4% vs. 7.4% vs. 14.0%, p = 0.016) (Table 5).

Kaplan-Meier survival plot showed that patients with systolic BP ≥ 160 mmHg had the highest total cardiovascular events, followed by patients with systolic BP < 120 mmHg and then patients with systolic BP 140–159 mmHg; patients with systolic BP 120–139 mmHg had the lowest total cardiovascular events (p < 0.001) (Fig. 1C).

A J-shaped curve was shown for the occurrence of the total cardiovascular events, with increased risk at low and high systolic BP values (Fig. 2C).

Cox regression showed that patients with systolic BP < 120 mmHg (HR, 1.634; 95% CI, 1.172–2.278, p = 0.004) and systolic BP ≥ 160 mmHg (HR, 2.546; 95% CI, 1.556–4.167, p < 0.001) had increased risk of total cardiovascular events compared to those with systolic BP 120–139 mmHg. Multivariate analysis showed that patients with systolic BP < 120 mmHg (HR, 1.648; 95% CI, 1.177–2.308, p = 0.004) and systolic BP ≥ 160 mmHg (HR, 2.518; 95% CI, 1.528–4.149, p < 0.001) had increased risk of total cardiovascular events compared to those with systolic BP 120–139 mmHg (Table 6).

### Clinical outcomes of the patients in 24 months according to diastolic blood pressure

In 24 months, both the lowest and highest diastolic BP subgroups had higher total cardiovascular events (diastolic BP < 70 mmHg vs. 70–79 mmHg vs. 80–89 mmHg vs. ≥90 mmHg = 15.8% vs. 9.7% vs. 11.5% vs. 14.6%, p = 0.016) (Table 5).

Kaplan-Meier survival plot showed that patients with diastolic BP < 70 mmHg had the highest total cardiovascular events, followed by patients with diastolic BP ≥ 90 mmHg and then patients with diastolic BP 80–89 mmHg; patients with diastolic BP 70–79 mmHg had the lowest total cardiovascular events (p = 0.017) (Fig. 1D).

A J-shaped curve was shown for the occurrence of the total cardiovascular events, with increased risk at low and high diastolic BP values (Fig. 2D).

Cox regression showed that patients with diastolic BP < 70 mmHg (HR, 1.671; 95% CI, 1.192–2.341, p = 0.003) had increased risk of total cardiovascular events compared to those with diastolic BP 70–79 mmHg. Multivariate analysis showed that patients with diastolic BP < 70 mmHg (HR, 1.590; 95% CI, 1.125–2.247, p = 0.009) had increased risk of total cardiovascular events compared to those with diastolic BP 70–79 mmHg (Table 6).

## Discussion

The main findings of our study were that (1) CAD patients with both achieved systolic BP ≥ 160 mmHg and achieved systolic BP < 120 mmHg had increased total cardiovascular events in 12 months and 24 months follow-up, and (2) CAD patients with achieved diastolic BP < 70 mmHg had increased total cardiovascular events in 24 months follow-up. The findings support the J-curve phenomenon of BP in ethnic Chinese stable CAD patients.

The concept of “J-curve phenomenon” has been noted for decades. Stewart IM found that the relative risk of myocardial infarction in patients with post-treatment diastolic BP < 90 mmHg was more than five times that in patients with diastolic BP 100–109 mmHg^12^. Cruickshank JM was the first to report the “J-curve phenomenon” in which a J-shaped relation was noted between diastolic BP during treatment and myocardial infarction, and with the lowest point of diastolic BP (the J point) between 85 and 90 mmHg^13^. In the ACCOMPLISH trial^14^, major cardiovascular events were significantly lower in those with systolic BP < 140 mmHg and <130 mmHg than those with BP > 140 mmHg. The incidence of composite coronary events (myocardial infarction, hospitalized angina, or sudden death) but not stroke was higher in those with systolic BP < 120 mmHg compared to those with systolic BP < 130 mmHg.

Some studies do not support the J-curve phenomenon. In the substudy of CAMELOT trial^15^, the most favorable rate of progression of coronary atherosclerosis observed by intravascular ultrasound occurred in subjects with a sustained BP < 120/80 mmHg. However, patients who had undergone PCI or had an angiographic diameter stenosis of >50% were excluded from this trial. Although the SPRINT trial supported more aggressive BP reduction for cardiovascular protection, only 16.7% of the patients had clinical cardiovascular diseases^4^.

Furthermore, there is new evidence about J-curve phenomenon of BP in general cohorts. Low diastolic BP was noted to be associated with subclinical myocardial damage and coronary heart disease events, especially in those with diastolic BP below 60 mmHg^16^. In a population of more than 1 million Korean individuals who participated in routine medical examinations, J-curve phenomenon was noted between systolic BP and vascular mortality, which reached a nadir at ≈100 mmHg. Systolic BP < 90 mmHg may portend death from vascular disease, particularly from ischemic heart disease^17^.

In addition to the CLARIFY trial^10^, J-curve phenomenon of BP in CAD patients was also supported by other studies^18–21^. In the post hoc analysis of the INVEST study^18, 19^, the J-shaped relationship was noted between BP and the primary outcome, all-cause death and myocardial infarction, particularly for diastolic BP with a nadir at 119/84 mmHg. In the TNT trial^20^, the relationship between BP and the primary outcome followed a J-curve even after adjusting for baseline covariates, treatment effect, and low-density lipoprotein cholesterol levels. Patients with lower systolic BP (<110–120 mmHg) or diastolic BP (<60–70 mmHg) have increased risks of future cardiovascular events (except stroke). In the PROVE IT-TIMI 22 trial^21^, a J-curve association was noted between BP and the risk of future cardiovascular events, and the study suggested that too low of a BP (especially <110/70 mmHg) may be dangerous. The findings of our study further supported the J-curve phenomenon in a Taiwanese population of CAD patients, in which lower achieved systolic BP (<120 mmHg) or diastolic BP (<70 mmHg) had increased risks of total cardiovascular events.

According to the recent statement by the American Heart Association, American College of Cardiology, and American Society of Hypertension^22^, BP target for patients with hypertension or CAD is <140/90 mmHg. BP < 130/80 mmHg may be appropriate, especially in those with a history of a previous myocardial infarction or stroke, or at high risk for developing either. After the SPRINT trial, there are suggestions that these numbers need to be revised^23^. According to the recent guideline by the European Society of Cardiology^24^, a target systolic BP < 120 mmHg may be considered in some patients if they are at high-risk and tolerate multiple BP lowering drugs. However, the findings of our study, together with CLARIFY registry, raise concerns that systolic BP less than 120 mmHg and diastolic BP less than 70 mmHg may be associated with adverse cardiovascular outcomes in patients with stable CAD. Furthermore, HOPE-3 trial showed that BP treatment does not always confer to lower rates of major cardiovascular events^25^.

Although hypertension guidelines suggest BP targets in different populations, the hypertension control rate is still unsatisfied. The Taiwanese Secondary Prevention for Patients with AtheRosCLErotic Disease (T-SPARCLE) Registry was a multicenter observational registry conducted in 14 hospitals in Taiwan^26^. A total of 3,316 outpatients who had established cerebrovascular disease, CAD, or both were recruited. Overall, only 55.9% of patients could achieve BP < 140/90 mmHg for nondiabetic patients and <130/80 mmHg for diabetic patients. In the current study, there were 733 diabetic patients and 1,312 non-diabetic patients. Among these patients, 270 diabetic patients (36.8%) achieved BP < 130/80 mmHg, and 970 non-diabetic patients (73.9%) achieved BP < 140/90 mmHg. Overall, 1,240 of 2045 patients (60.6%) achieved BP < 140/90 mmHg for nondiabetic patients and <130/80 mmHg for diabetic patients. This finding was compatible with the data of the T-SPARCLE Registry. Current hypertension guidelines in Taiwan suggest BP target <130/80 mmHg for CAD patients with or without diabetes^27^. Only 879 patients (43.0%) achieved BP < 130/80 mmHg in our registry, suggesting that more efforts are still required for hypertension management in patients with CAD.

There are some possible mechanisms for the J-curve phenomenon of BP in CAD patients. First, perfusion of the heart might be compromised at too low diastolic BP since the heart is perfused during diastole. For CAD patients, a coronary stenosis will lower the perfusion pressure in the downstream territory, and the autoregulation will also be altered. Therefore, there is a higher possibility of myocardial ischemia when lowering diastolic BP^16, 28^. Second, BP changes continuously from systole to diastole. It is impossible to lower systolic BP without influencing diastolic BP. In order to achieve intensive systolic BP reductions, the diastolic BP may simultaneously become too low, especially in elderly patients with wide pulse pressures.

### Study limitations

There were some limitations in the current study. First, the sample size was relatively small; therefore, we only divided CAD patients into 4 systolic or diastolic BP subgroups. Although we did adjust multiple confounding factors and demonstrated the clear J-curve phenomenon in both systolic and diastolic BP within 24 months follow-up, we did not perform further subgroup analyses of age, gender, or other comorbidities. Further studies with larger sample sizes are still needed. Second, in this study, all the patients were stable during enrollment and followed up regularly for clinical events in the out-patient clinics of the medical centers or teaching hospitals. Their medications may have been adjusted by the specific cardiologists during follow-up according to individual BP changes. Thus, the potential effects of different antihypertensive drugs on clinical outcomes could not be well addressed. Third, most of the events were cardiac events, and the number of strokes was relatively few. We could not determine whether there were different impacts of BP on CAD and stroke, which have been noted in other studies^7, 14, 20^. Fourth, the follow-up durations were limited to only 12 months and 24 months. Although we observed the J-curve phenomenon within 24 months, further studies with long-term follow-ups are still needed. Finally, the BP measurement in our study included only office BP. Further studies to assess ambulatory BP or other BP measurements should be considered to offer more information about BP management in CAD patients.

## Conclusion

In a cohort of ethnic Chinese patients with stable CAD in Taiwan, we found the J-curve phenomenon of BP in both 12- and 24-month follow-up. CAD patients with achieved systolic BP < 120 mmHg and ≥160 mmHg or diastolic BP < 70 mmHg had increased cardiovascular events in 24 months. While the response to the changes of BP may vary for clinical outcomes, our findings may provide a rationale to justify whether the BP goals suggested by recent clinical studies in western cohorts, such as SPRINT trial, should be extended to other population cohorts. Aggressive BP control in CAD patients requires caution.
